# Urban energy load dataset of Chattogram: Peak and off-peak variability insights

**DOI:** 10.1016/j.dib.2025.112245

**Published:** 2025-11-06

**Authors:** Arnab Barua, Shah Nawaz Haider, Steve Austin, Sarowar Morshed Shawon

**Affiliations:** a Department of Electrical and Electronic Engineering, University of Science and Technology Chittagong, Chattogram 4202, Bangladesh; b Department of Computer Science and Engineering, University of Science and Technology Chittagong, Chattogram 4202, Bangladesh

**Keywords:** Urban energy load, Load forecasting, Chattogram, Bangladesh

## Abstract

This article presents a comprehensive dataset regarding the power demand for the period from January 1, 2021 to June 30, 2023, for the Chattogram region of Bangladesh. Chattogram is one of the country’s fastest-growing industrial and residential regions where the demand for electricity and power is rapidly increasing. This is proportionate to urbanization, industries and residential power consumption. This dataset captures demand on a daily basis for the Day Peak (9AM-5PM), Evening Peak (6PM-9PM) and Off-Peak (10PM-8AM) hours and captures demand for industrial, residential and basal load during each period. To enhance the analysis, demand was further correlated to weather data obtained from the NASA POWER database which included temperature, dew-point, wet bulb temperature, skin temperature, humidity, air moisture and precipitation. Correlation analysis indicates strong, demand predictors, which are thermal features and specific humidity demand, whereas, relative humidity and precipitation were found to affect demand poorly. Integrated and automated data collection was performed via substation equipment resulting in cleaned and normalized data for model processing. This dataset allows the use of machine learning to accurately forecast real-world scenarios. By combining meteorological and load data, this resource supports research on energy efficiency, climate resilience and grid reliability in rapidly urbanizing regions.

Specifications Table

**Table:** 

Subject	Computer Sciences
Specific subject area	Data on electricity load demand along with meteorological data for Chattogram, Bangladesh.
Type of data	Tabular/Numerical
Data collection	The daily Day Peak, Evening Peak and Off-Peak data was collected manually from the regional office of PGCB, based on the time of the day. Day Peak consists of load data from 9 AM to 5 PM, Evening Peak from 6 PM to 9 PM and Off-Peak from 10 PM to 8 AM.
Data source location	The data was collected from the regional substation of PGCB, that covered the city of Chattogram
Data accessibility	Repository name: Mendeley Data Data identification number: https://doi.org/10.17632/djdg2d3rbz.1 Direct URL to data: https://data.mendeley.com/datasets/djdg2d3rbz/1
Related research article	S. M. Shawon et al., “Hybrid CNN-LSTM model for urban energy load forecasting with IGA-XAI for smart grids: Peak and off-peak variability insights,” *Results Eng.*, vol. 28, p. 107,245, Dec. 2025, doi:10.1016/j.rineng.2025.107245.

## 1 Value of the Data

• The dataset provides a detailed multi-year record (January 2021-June 2023) of electricity demand in Chattogram, Bangladesh, across three distinct time intervals: Day Peak, Evening Peak, and Off-Peak. These periods reflect industrial, residential, and baseline consumption patterns, respectively, offering a fine-grained view of urban load variation.
• It integrates meteorological parameters such as temperature, dew point, wet bulb temperature, earth skin temperature, humidity, and precipitation from the NASA POWER database, enabling analysis of weather-driven variations in energy demand without relying solely on national-level aggregates.
• The dataset supports the development, training, and evaluation of advanced forecasting models, including deep learning architectures such as CNN-LSTM, to capture nonlinear and temporal dependencies in energy consumption.
• Its standardized structure allows integration with regional or national datasets, facilitating comparative research on grid management, demand response, and climate-related impacts on urban energy use.
• The dataset underpins ongoing research on energy forecasting in Chattogram and complements the related study by Shawon et al. [1], enhancing understanding of peak and off-peak variability in urban load behaviour.

## 2 Background

The Chattogram region has emerged as one of Bangladesh's major industrial and commercial hubs, with rapid urbanization and expanding residential activity driving electricity demand growth [2]. To ensure stability and efficiency in supply, the Power Grid Company of Bangladesh (PGCB) provided data from January 1, 2021, to June 30, 2023, documenting electricity consumption across three primary intervals: Day Peak (9 AM-5 PM), Evening Peak (6 PM-9 PM), and Off-Peak (10 PM-8 AM); capturing industrial, residential, and baseline loads [3].

To enhance the demand analysis, meteorological data from the NASA POWER (Prediction of Worldwide Energy Resources) database were incorporated, including temperature, humidity, precipitation, and related thermal variables. Prior research highlights temperature as the main driver of electricity use in hot climates with high cooling demand [[4], [5], [6]]. In the short term, demand is affected by humidity and precipitation, while wet bulb and earth skin temperatures relate to thermal stress and energy use [7,8]. Overall, these variables bolster the argument regarding their role in urban energy use.

The integration of load and climate data enables exploration of weather-demand interactions in urban contexts. This dataset uniquely supports the modelling of nonlinear temporal dynamics in forecasting applications and provides a foundation for climate-resilient energy planning in Chattogram. It complements the related study by Shawon et al. [1], where the dataset enhanced hybrid CNN-LSTM modelling performance in capturing spatio-temporal variability in electricity consumption. The article highlights the importance of peak and off-peak variability insights in practical load data, that is seen in actual power systems.

## 3 Data Description

The data presented in- “Urban Energy Load Dataset of Chattogram: Peak and Off-Peak Variability Insights” has been collected from the Chattogram office of the PGCB covers the time period of January 1, 2021, to June 30, 2023 [9]. It records the daily electricity demand for one of the most economically important regions of the country, with local time-stamps. In contrast to aggregated national datasets, this captures demand for Chattogram closely, considering the structure of both its industries and the residential electricity consumption. To strengthen the dataset for forecasting, meteorological variables from the NASA POWER database were incorporated, which allowed for a comprehensive evaluation of the climate demand variables. The subsequent subsections describe the dataset, including and not limited to the correlation analysis of the primary features and the normalization of the dataset for modelling.

**Fig. 1:**
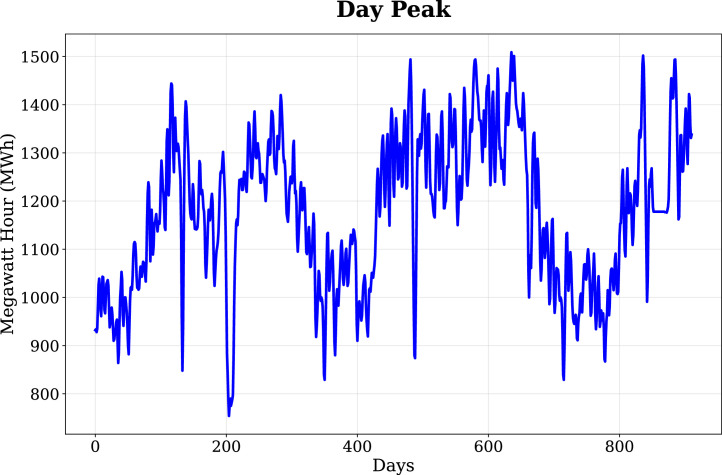
Day Peak load profile, showing daily energy demand dynamics and variability patterns across the study period.

**Fig. 2:**
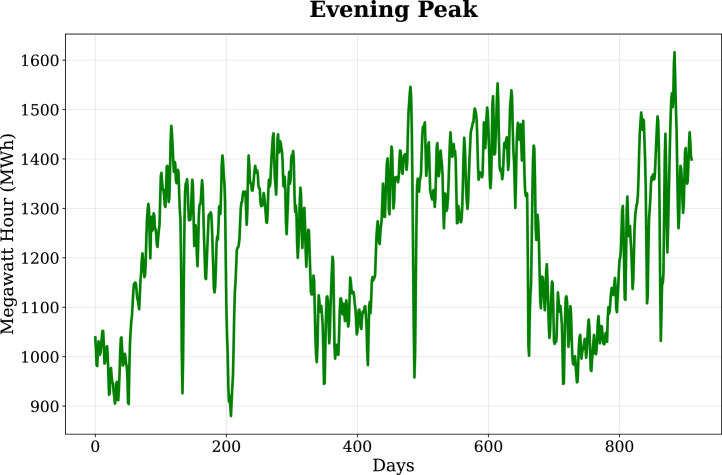
Evening Peak load profile, showing daily energy demand dynamics and variability patterns across the study period.

**Fig. 3:**
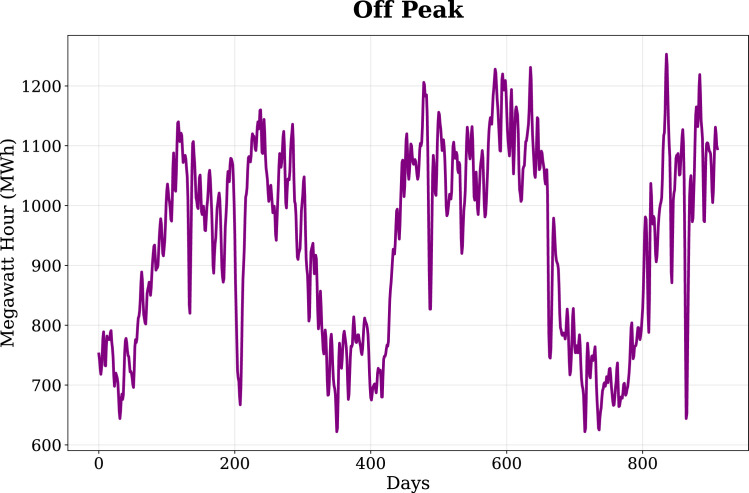
Off-Peak load profile, showing daily energy demand dynamics and variability patterns across the study period.

To analyze distinct patterns in electricity usage, the data was structured around 3-time intervals: Day Peak (9 AM-5 PM), Evening Peak (6 PM-9 PM) and Off-Peak (10 PM-8 AM). These intervals align with the electricity consumption patterns during the Day Peak, Evening Peak and Off-Peak periods in Chattogram, considering the Day Peak is mostly influenced by commercial and industrial activities, the Evening Peak by household demand and the Off-Peak periods baseline consumption which ensures essential services are delivered. The Day Peak load profile, illustrating daily energy demand dynamics and variability patterns across the study period, is presented in Fig. 1. Similarly, the Evening Peak load profile, capturing household and mixed-use demand patterns, is shown in Fig. 2, while the Off-Peak load profile, reflecting baseline and essential service demand, is depicted in Fig. 3. The structure of the dataset, including column names, data types, measurement units, and example values, is summarized in Table 1.

**Table 1:** Parametric Description of the Load and Weather Dataset.

Column Name	Data Type	Unit	Description	Sample Value
**Date**	Numerical	-	Observation date in MM/DD/YYYY format	01/01/2021
**Temperature**	Numerical	°C	Average ambient air temperature for the day	18.76
**Dew/Frost Point**	Numerical	°C	Temperature at which dew or frost forms	14.99
**Wet Bulb Temperature**	Numerical	°C	Temperature considering humidity and evaporation	16.87
**Earth Skin Temperature**	Numerical	°C	Ground or surface temperature measured near the soil	18.62
**Min Temperature**	Numerical	°C	Minimum temperature recorded during the day	14.3
**Max Temperature**	Numerical	°C	Maximum temperature recorded during the day	23.85
**Specific Humidity**	Numerical	g/kg	Mass of water vapor per kilogram of air	10.56
**Relative Humidity**	Numerical	%	Ratio of current humidity to the maximum possible humidity at that temperature	79.94
**Precipitation**	Numerical	mm	Amount of rainfall or precipitation measured for the day	0
**Day Peak**	Numerical	kWh (Load)	Electricity load during Day Peak hours (9 AM – 5 PM)	932
**Evening Peak**	Numerical	kWh (Load)	Electricity load during evening peak hours (6 PM – 9 PM)	1039
**Off Peak**	Numerical	kWh (Load)	Electricity load during Off-Peak Hours (10 PM – 8 AM)	752

As shown in Table 1, the dataset provides a clear linkage between climatic factors and hourly load variations, serving as a valuable basis for energy forecasting and demand-side management analyses.

Understanding the influence of various climatic factors on electricity usage required the addition of other weather phenomenon data, including temperature, dew/frost point, wet bulb temperature, earth skin temperature, minimum and maximum temperature, relative humidity, specific humidity and precipitation. Prior studies by Ratanawaraha et al. [10] and Das et al. [11] have discussed temperature as the primary determinant of electricity demand, particularly during peak cooling periods, however humidity and precipitation also influence electricity consumption.

**Fig. 4:**
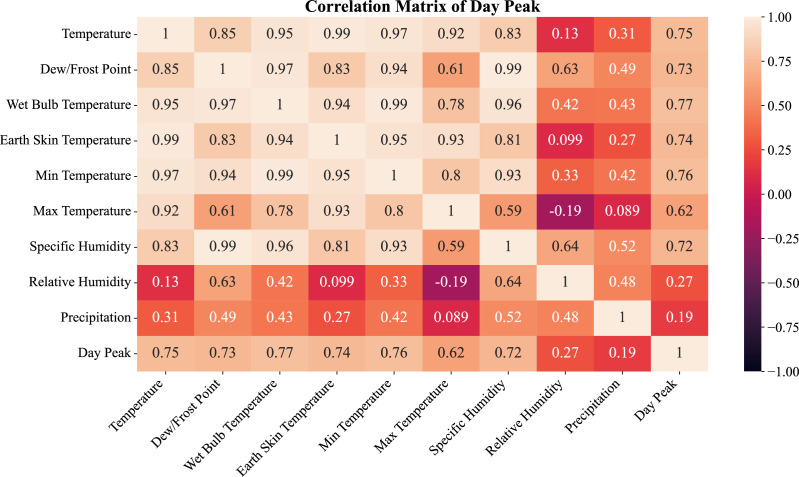
Feature Correlation for Day Peak load profile.

The positive correlations highlighted in these analyses are also supported in Fig. 4. During peak weekday daytime periods, the most significant correlations are between load demand and the other thermal variables (e. g. temperature, dew point and earth skin temperature). The correlation with specific humidity (*r* = 0.72) also suggests significant relationships with other humidity related variables. One the other hand, correlation with relative humidity (*r* = 0.27) and with precipitation (*r* = 0.19) are low which suggest that humidity and precipitation are hardly affecting the load demand directly at peak periods.

**Fig. 5:**
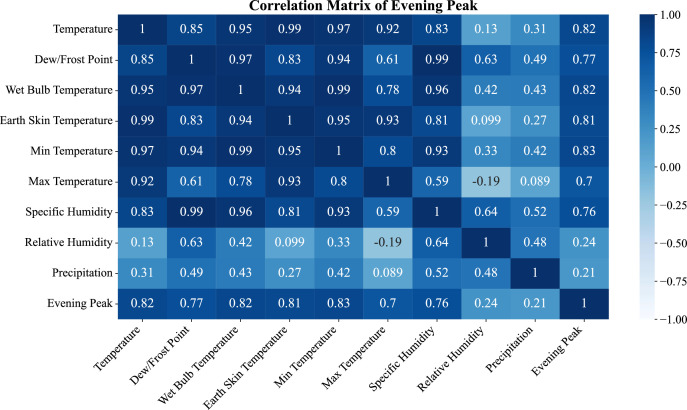
Feature Correlation for Evening Peak load profile.

The evening peak (Fig. 5) also highlighted the other variable correlations. Here the other thermal variables (ambient, skin and dew point temperatures) have elevated correlations (e. g. air temperature *r* = 0.82). Specific humidity (*r* = 0.76) and minimum temperature (*r* = 0.79) remain key variables and there is again weak correlation with relative humidity (*r* = 0.21) and precipitation.

**Fig. 6:**
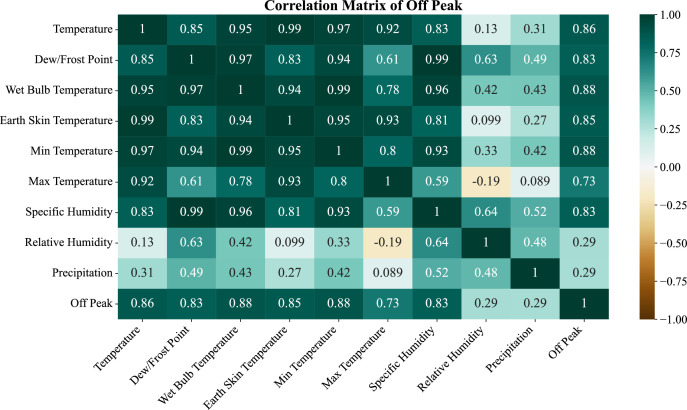
Feature Correlation for Off-Peak load profile.

The Off-Peak period (Fig. 6) showed a similar correlation pattern with ambient temperature (*r* = 0.86) and dew point temperature (*r* = 0.88) being the strongest. The other thermal variables combined with specific humidity showed similar correlations with minimum temperature (*r* = 0.80). As noted, relative humidity (*r* = 0.29) and precipitation again had low influence, further supporting the primary linkage of the other variables combined with temperature during these periods.

These results demonstrate that temperature-related factors and humidity dominate demand across all time segments, justifying the use of advanced ML and Deep Learning (DL) methods capable of capturing nonlinear and temporal relationships that extend beyond simple linear associations.

Overall, the dataset presents a balanced and detailed view of electricity demand in Chattogram alongside critical weather indicators. The three distinct peak and off-peak intervals provide insight into distinct consumption behaviors, while correlation analysis highlights the strong role of thermal and humidity-related factors in shaping demand. Through normalization, the dataset has been prepared for effective application in statistical, ML and DL models, and capturing complex spatial and temporal dependencies of load data.

## 4 Experimental Design, Materials and Methods

The data was collected form the Chattogram Divisional office of PGCB, starting from 1st January 2021 to 30th June 2023. The data was collected manually and later shifted to a hybrid of physical and autonomous data collection through the introduction of Sub-station Automation System by PGCB. This enabled the data collection procedure become more automated, though manual intervention was required time-to-time, as the system was still under adoption phase.

**Fig. 7:**
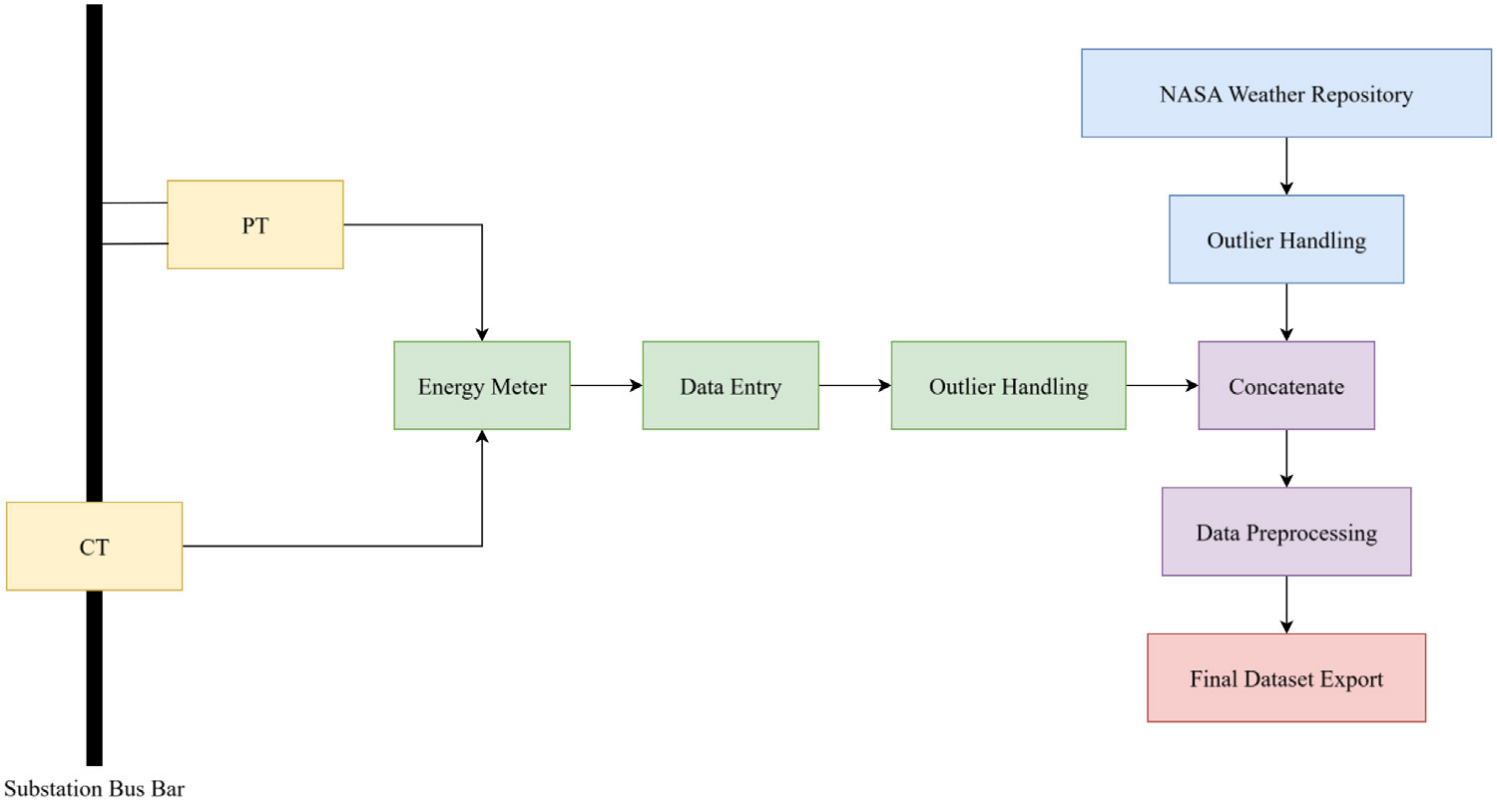
Data Collection and Processing Methodology.

Fig. 7 illustrated the entire data collection and processing architecture in a simplified workflow diagram. The high voltage and current was converted through potential transformer (PT) and current transformer (CT) to suitable operational levels for the energy meter. The outliers and missing values were identified and processed through rolling-mean. These outliers were, for most cases, typographic error, or mis-entries.

Outliers and missing values were identified and corrected using a rolling-mean filtering approach. In most cases, the outliers resulted from typographical errors or data entry inconsistencies. A threshold-based validation check was also applied to ensure that values deviating significantly (beyond 20 % of standard deviation range) from the local mean were appropriately adjusted by using the rolling mean of 5 adjacent values. Overall, only 2 missing data along with 5 outliers were identified.

Meteorological data was then collected from NASA POWER, which included an extensive feature set to the data like- temperature, Dew/Frost Point, Wet Bulb Temperature, Earth Skin Temperature, Min Temperature, Max Temperature, Specific Humidity, Relative Humidity and Precipitation. This collection of features allows the data to be used by any training model to accurately forecast electricity load across the city of Chattogram.

Overall, the data collection procedure was done keeping research implication in mind. The three distinct patterns reveal variations in consumption behaviors, while the correlation analysis emphasizes the significant influence of thermal and humidity-related factors on demand.

## Limitations

The dataset, while comprehensive, has some inherent limitations. In the early stages of the project, the reliance on manual entry introduced errors, that were later corrected with rolling-mean filtering and validation. The meteorological variables were obtained through the NASA POWER, which contains satellite-derived estimates and not local ground measurements, and some of those features may lessen the spatial accuracy of the estimates, as seen in the Fig. 4, Fig. 5, Fig. 6. The temporal coverage is limited to January 2021–June 2023, constraining analysis of long-term demand shifts. Furthermore, the data contains aggregated regional demand and does not include intra-division disaggregation which obscures insights relating to micro-level consumption. The gaps and inconsistencies stemming from the transitional deployment of PGCB’s Sub-station Automation System were also corrected through pre-processing. In spite of these limitations, the dataset is still robust enough to undertake sophisticated modelling and forecasting.

## Ethics Statement

The authors confirm that they have read and comply with the ethical requirements for publication in Data in Brief. This work does not involve human subjects, animal experiments, or data collected from social media platforms. The dataset comprises electricity demand records obtained from the Chattogram Divisional office of the Power Grid Company of Bangladesh (PGCB) and meteorological variables from the publicly accessible NASA POWER database. All data were collected, processed and reported in accordance with institutional and publisher guidelines.

## Credit author statement

**Arnab Barua:** Data curation, Writing - original draft, Writing - review & editing; **Shah Nawaz Haider**: Methodology, Software, Data curation, Validation, Visualization, Resources; **Steve Austin:** Investigation, Data curation, Formal analysis, Writing - original draft, Writing - review & editing; **Sarowar Morshed Shawon:** Conceptualization, Data Curation, Writing - Review & Editing, Project administration, Supervision

## Data Availability

Mendeley DataUrban Energy Load Dataset of Chattogram: Peak and Off-Peak Variability Insights (Original data)
